# Mesophilic Semi-Continuous Anaerobic Digestion of Strawberry Extrudate Pretreated with Steam Explosion

**DOI:** 10.3390/foods9121887

**Published:** 2020-12-17

**Authors:** Juan Cubero-Cardoso, Andrés Muñoz-Arjona, Ángeles Trujillo-Reyes, Antonio Serrano, Bernabé Alonso-Fariñas, Guillermo Rodríguez-Gutiérrez, Juan Urbano, Rafael Borja, Fernando G. Fermoso

**Affiliations:** 1 Instituto de Grasa, Spanish National Research Council (CSIC), Ctra. de Utrera, km. 1, 41013 Seville, Spain; juan.cubero@ig.csic.es (J.C.-C.); atrujillo@ig.csic.es (Á.T.-R.); antonio.serrano@ig.csic.es (A.S.); guirogu@ig.csic.es (G.R.-G.); rborja@ig.csic.es (R.B.); 2 Department of Chemistry, Faculty of Experimental Sciences, University of Huelva, 21007 Huelva, Spain; juan.urbano@dqcm.uhu.es; 3 Departamento de Ingeniería Química y Ambiental, Escuela Técnica Superior de Ingeniería, Universidad de Sevilla, Camino de los Descubrimientos s/n, 41092 Seville, Spain; andmunarj@gmail.com (A.M.-A.); bernabeaf@us.es (B.A.-F.); 4 School of Civil Engineering, The University of Queensland, Campus St. Lucia-AEB Ed 49, St. Lucia, QLD 4067, Australia

**Keywords:** strawberry extrudate, anaerobic digestion, economic assessment, steam explosion, phenol recovery, valorisation

## Abstract

The production of strawberry concentrate produces a side stream after extrusion that is commonly landfilled. This strawberry extrudate (SE), of lignocellulosic character, contains valuable bioactive compounds such as sugars and phenols. Thermal treatments, such as steam explosion, are currently used for the valorisation of agricultural lignocellulosic wastes due to their ability to impact the structure of the lignocellulose and hemicellulose present in these wastes, favouring the disruption of fibrous material. Steam explosion has already been shown as a promising technology for phenol recovery from SE. Biogas is an additional valuable resource that might be produced from thermally pretreated and de-phenolised SE. This study assessed the influence of a steam-explosion pretreatment and the subsequent recovery of phenolic compounds from the long-term operation of a semi-continuous anaerobic digester of pretreated SE. The anaerobic digestion of SE steam exploded at 220 °C for 5 min and de-phenolised was stable at an OLR of 0.5 g of volatile solids (VS)/(L·d), which permitted a specific production rate of 135 ± 11 mL of CH_4_/(g of VS d). The system was not able to operate at an OLR of 1 g of VS/(L·d), which resulted in a failure of the process. Despite the inhibition threshold of phenolic compounds not being achieved, the inhibition of the anaerobic digestion process at an OLR of 1 g of VS/(L·d) was most likely due to the overloading of the system. This was indicated by the accumulation of soluble organic matter and volatile fatty acids. The increase in the propionic acid concentration up to 1300 mg/L when operating at OLRs higher than 0.5 g of VS/(L·d) could be the main factor responsible for the inhibition. An economic evaluation showed that the proposed approach (steam explosion, phenol recovery, and anaerobic digestion) would offer positive benefits, taking into account the high phenolic recovery (0.90 g of gallic acid equivalents/kg of extrudate) and the low sales price of the phenol extract, i.e., EUR 0.610/g of gallic acid equivalents, needed to reach zero net profit.

## 1. Introduction

Strawberries contain a wide variety of nutrients and phytochemicals, which are of great interest because they are beneficial for avoiding or preventing different cardiovascular, cancerous, and neurological diseases, among others [1,2]. Given the excellent nutritional properties of the strawberry, most of the strawberries produced are distributed and sold in the fresh market. In recent years, an important part of this production has been the production of strawberry concentrate for products such as jams, yogurts, ice creams, juices, etc. [2]. During the industrial process for obtaining strawberry concentrate, the strawberries are extruded through sieves with different mesh sizes, in such a way that a residual fraction, formed by the fibrous part and the achenes, is retained, called the strawberry extrudate (SE) [3]. SE is, to some extent, used as animal feed, but most of it is landfilled [4]. Alternatives for the management of the SE are necessary to avoid severe environmental impacts caused by landfilling, such as negative effects on agricultural soil quality, atmospheric contamination, and/or the polluting of aquatic ecosystems.

Cubero-Cardoso et al. [4] proposed the recovery of bioactive compounds from SE using heat treatments. These bioactive compounds can be used in the pharmaceutical, food, and/or cosmetic industries [2,3]. The bioactive compounds from SE include ascorbic acid, anthocyanins, ellagitannins, different vitamins, carotenoids, folic acid, and flavonoids [1]. Heat treatments move these compounds to a liquid phase, where they can be easily recovered [5]. One heat treatment widely used for lignocellulose material is steam explosion [6]. The steam-explosion treatment mainly affects the solubilization of the cellulose and lignin in the SE [6,7]. Under steam-explosion conditions, the lignin is highly degraded, and the hemicellulose is hydrolysed into its component sugars [3]. Cubero-Cardoso et al. [4] showed that the steam-explosion treatment of SE at 220 °C allowed the recovery of up to 2 g of gallic acid equivalents per kg of residual SE.

Cubero-Cardoso et al. [4] also studied the anaerobic digestion of the steam-exploded and de-phenolised SE in batch mode. The anaerobic digestion of lignocellulosic materials, such as SE, is a promising alternative to valorising these wastes because this treatment allows generating renewable and sustainable energy in the form of biogas [8,9]. The pretreatment of SE, such as steam explosion, could be a good complement for obtaining phenolic compounds with health benefits and, at the same time, being able to improve their biodegradability by anaerobic digestion through the degradation of recalcitrant components [10,11,12].

The present work aimed to assess the long-term, semi-continuous mesophilic anaerobic digestion of a de-phenolised SE previously treated with steam explosion. Long-term, semi-continuous operation is crucial for studying process efficiency and reducing the risk of destabilization. The anaerobic digestion process was carried out over a long operational period (312 days), assessing different organic loading rates (OLRs), which were equivalent to several hydraulic retention times (HRTs).

## 2. Materials and Methods

### 2.1. Strawberry Extrudate and Inoculum

The SE was provided by the company Hudisa S.A, located in Huelva, Spain (37.281813, −7.239095, Huelva, Spain). The SE was immediately stored at −20 °C after collection to avoid the uncontrolled fermentation of the substrate.

The anaerobic inoculum used in the anaerobic digestion process was obtained from the industrial anaerobic reactors located in the “Copero” wastewater treatment plant, located in Sevilla, Spain (37.309795, −5.986825, Dos Hermanas, Spain). The main chemical characteristics of the anaerobic inoculum were pH = 7.1 ± 0.1; alkalinity = 4500 ± 65 mg of CaCO_3_/L; total solids (TS) = 21,100 ± 92 mg/L; volatile solids (VS) = 13,350 ± 194 mg/L; total chemical oxygen demand (CODt) = 18,870 ± 630 mg of O_2_/L; soluble COD (CODs) = 520 ± 8 mg of O_2_/L; total phenols = 100 ± 1 mg of gallic acid equivalents/L; and total sugars = 20 ± 1 mg of glucose equivalents/L.

### 2.2. Steam-Explosion Treatment and Extraction of Phenolic Compounds

The steam-explosion treatments were performed in a 2 L-capacity, pilot-scale reactor (Nusim, S.A., Madrid, Spain). The treatments with strawberry extrudate were carried out twice throughout the experiment; the first time, 8.25 kg of SE was treated, and the second time, 4.93 kg of SE was processed. These were performed at a temperature of 220 °C and 25 kg/cm^2^ pressure for 5 min. The amounts of steam consumed in both treatments were 6.71 and 2.64 kg of steam/kg of SE, respectively. After each treatment, the treated waste was centrifuged at 4700× *g*/1450 rpm (Centrifuge Comteifa, S. L., Barcelona, Spain) to separate a liquid phase (LP) and a solid phase (SP).

The extraction of the phenolic compounds was carried out with a column, 4.5 cm in diameter and 140 cm in height, filled with 1 L of adsorbent resin Amberlite XAD-16, which was described by Cubero-Cardoso [4]. The extraction of the phenolic compounds retained in the Amberlite was carried out according to Fernández-Bolaños et al. [13]. The physicochemical characterization of untreated SE and different phases resulting from both steam explosion treatments and subsequent extraction of phenolic compounds from SE is shown in Table 1.

### 2.3. Anaerobic Digestion Set-Up and Experimental Procedure

Anaerobic digestion reactors in triplicate were used. The three reactors were initially inoculated with 10 g of VS/L of inoculum. The reactors had a total capacity of 2.0 L, with a working volume of 1.7 L, and were continuously stirred by means of a stirrer (KMO 2 basic model, IKA-WERKE, Staufen, Germany). The operation temperature (35 °C) was maintained by a thermostatic chamber. The volume of methane was measured daily after CO_2_ removal with tightly closed bubblers containing a (3-N) NaOH solution. The gas volume was measured by liquid displacement and expressed under standard temperature and pressure conditions (25 °C and 1 atm).

The anaerobic digestion of the phases obtained after the steam-explosion treatments at 220 °C and subsequent extraction of the phenolic compounds (SP + DLP) was carried out for 312 days, after an adaptation period of about 30 days, in which mixtures of a synthetic solution and SE were used (Table 1). The anaerobic digestion experimental study was carried out by using different organic load rates (OLRs), with three hydraulic retention times (HRTs) (Table 2). Additionally, the substrate resulting from the first steam-explosion treatment was used from the first day to the 230th day, while the SE from the second steam-explosion treatment was employed from the 231st day to the end of the study (Day 312).

### 2.4. Chemical Analyses

To measure the water-soluble compounds of the SE and the solid phase (SP) after the steam-explosion treatments, an extraction process was carried out as explained in Cubero et al. [4]. To extract the soluble compounds from the liquid phase (LP), de-phenolised liquid phase (DLP), and effluents of the reactors, the samples were centrifuged and microfiltered with 0.45 µm nylon microfilters.

The pH, TS, VS, and alkalinity were analysed according to the standard methods of the American Public Health Association (APHA, 2017). The total chemical oxygen demand (COD_t_; mg of O_2_/L) was measured as explained in Cubero et al. [4]; the colorimetric standard method 5220D was used for the measurement of the soluble COD (CODs; mg of O_2_/L) [14]. Total phenols were measured, after extraction with a methanol/water solution (80:20) at 70 °C and subsequent filtration through 0.45 µm filters, by the Folin–Ciocalteu spectrophotometric method and expressed as mg of gallic acid equivalents/L [15,16]. Total sugars were measured by the anthrone colorimetric method, using a spectrophotometer (Biorad iMark Microplate Reader, Hercules, CA, USA), and expressed as mg of glucose equivalents/L [17]. The determination of volatile fatty acids (VFAs) was carried out using gas chromatography. More details about the VFA determination can be found in Cubero et al. [4].

### 2.5. Economic Assessment

An economic evaluation was carried out to calculate the minimum sale price of the extracted phenols that allows a positive economic balance. The case studied includes steam-explosion treatment, phenol extraction, and anaerobic digestion with the most stable OLR. The generated biogas is used for the simultaneous generation of electricity and heat. The net benefit of the different options was defined as the economic balance between the operational costs and income from sales. The minimum cost for the sale of the phenols extracted was calculated by imposing a value of zero on the net benefit and was expressed in EUR/g of gallic acid equivalents. The phenol extraction efficiency was 64%, which was obtained from the reduction of gallic acid observed in the SE during the experiments and its increase in the liquid phase after the extraction process with respect to untreated SE. The energy production was based on the methane production for Stage 2, i.e., 57–107 days and OLR = 0.5 g of VS/(L·d) (Table 2), and the use of a cogeneration biogas engine for electricity and thermal energy. The energy consumption for the steam explosion was based on the steam requirement, i.e., 6.71 kg of steam/kg of SE. More details about the previous considerations for the economic assessment can be found in Trujillo-Reyes et al. [11].

## 3. Results and Discussion

### 3.1. Methane Production Rate at Different Organic Loading Rates throughout the Experimental Period

**Figure 1:**
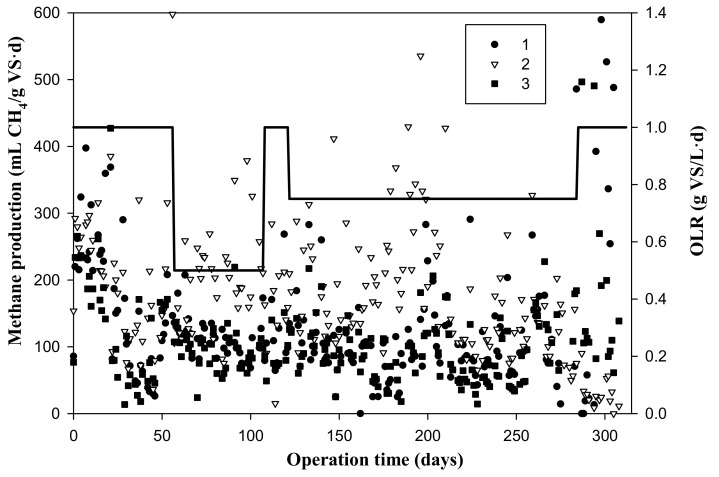
Variation of the methane production rates and organic loading rates (OLR, bold line) during the whole experimental time.

Figure 1 shows the variation of the daily methane production rate throughout the digestion time for the different OLRs tested. Table 3 summarizes the pH, alkalinity, TS, VS, CODs, total VFA, and total phenols of the effluents of the semi-continuous anaerobic digestion process for the steam-exploded SE, as well as the methane production rates and biodegradability achieved for the different organic loading rates (OLRs) tested over the five operational periods carried out. During the first 20 days, a methane production rate higher than 250 mL of CH_4_/(g of VS·d) was observed, a value that was not achieved again throughout the experiment. After these first 20 days, the reactors were destabilized, exhibiting a significant drop in the methane production rate. In Stage 1, i.e., 0–56 days and OLR = 1 g of VS/(L·d), the methane production rate presented a mean value of around 124 ± 46 mL of CH_4_/(g of VS d) and a biodegradability of 70% (Table 3). Given that the methane production rate was unstable in Stage 1 at OLR = 1 g of VS/(L·d), it was decided to decrease the organic loading rate to a value of 0.5 g of VS/(L·d). In Stage 2, i.e., 57–107 days and OLR = 0.5 g of VS/(L·d), the methane production rate observed was more stable, reaching a mean value of around 135 ± 11 mL of CH_4_/(g of VS·d), and the biodegradability was 53%. It can be seen that the biodegradability was 20% lower than that achieved in Stage 1, because the reactors were more stabilized, and they had been accumulating solids with low biodegradability since the beginning of the experiment. In Stage 3, i.e., 108–121 days, it was decided to again raise the OLR to 1 g of VS/(L·d) since in Stage 2, the reactor was stabilized. In Stage 3, the methane production rate presented a mean value of around 130 ± 10 mL of CH_4_/g of VS d and a biodegradability of 51%, which were very similar to those achieved in Stage 2. As can be seen in Figure 1, the reactors could not withstand an OLR = 1 g of VS/(L·d), so it was decided not to maintain this OLR over an operational period equivalent to three times the HRT and to quickly change to a lower load before the reactors could destabilize. In Stage 4, i.e., 122–284 days and OLR = 0.75 g of VS/(L·d), the methane production rate showed a mean value of around 129 ± 45 mL of CH_4_/g of VS d, and the biodegradability was 60%. In this Stage 4, it was decided to maintain this load for a longer time for the study of the stability of the OLR, and it was observed that the stability could only be maintained when the reactors were supplemented with alkalinity. Finally, in Stage 5, i.e., 285–310 days, the OLR was again augmented up to OLR = 1 g of VS/L d to finish observing that at this load, the reactors were not stable; the methane production rate presented a mean value of around 64 ± 43 mL of CH_4_/g of VS d, and the biodegradability was 48%. The characteristics of the substrate described in Table 1 indicate that the acidification of the reactors could occur, and therefore, an inhibition of methane production, due to the low pH value and the high sugar content, could take place [18]. Due to the instability observed in the methane production rates throughout the entire experimental period of the semi-continuous anaerobic digestion process, the highest methane production achieved in this process was lower than that achieved in the batch anaerobic digestion experiments for this steam-exploded waste after 24 days of digestion time, which was 430 mL of CH_4_/g of VS [4]. Siles et al. [19] reported that the methane production in the anaerobic digestion of strawberry extrudate, with similar conditions but without pretreatment, was 230 mL of CH_4_/g of VS at OLRs = 0.73 and 1.07 g of VS/(L·d). In another study, untreated strawberry residue obtained from rejections provided by a supermarket yielded a methane production rate of 231 mL of CH_4_/g of VS at an OLR = 4.4 g of VS/(L·d) [20]. Finally, a recent study of the anaerobic digestion of strawberry waste at a pH controlled at 7 reported a methane production of 353 ± 10 mL of CH_4_/g of VS [21], which was higher than the rates reported in the above-mentioned studies.

### 3.2. Stability and Organic Matter Soluble Concentration throughout the Experimental Time

**Figure 2:**
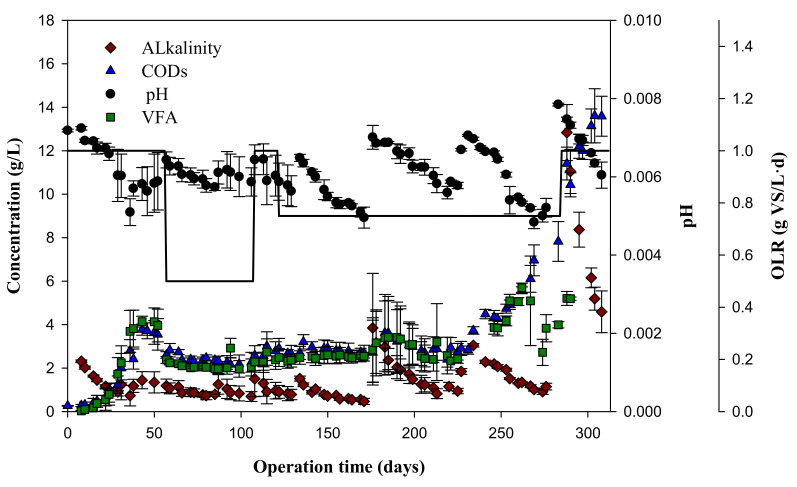
Variation of pH (●), alkalinity (♦), CODs (▲), and VFA (■) values, with their standard deviations, throughout the experimental period for the different organic loading rates (OLRs, bold line) assayed.

Throughout the experimental time, the pH, alkalinity (mg of CaCO_3_/L), CODs (mg of O_2_/L), and total VFAs (mg of O_2_/L) were determined to evaluate the process stability and the variation of organic matter with time in the semi-continuous anaerobic digestion process (Figure 2). As can be seen, the pH was only stabilized at an OLR = 0.5 g of VS/(L·d), in Stage 2. At OLRs higher than 0.5 g of VS/(L·d), the pH began to decrease, due to the pH of the added load always being less than 4.5 (Table 2). Therefore, for OLRs greater than 0.5 g of VS/(L·d), the alkalinity must be increased so that the reactors can buffer changes in pH. It can be seen in Stages 1 and 4 that the pH tended to decrease from pH 7.6–7.8, which can be considered as the optimal pH (Wheatley, 1990), to an acidification value that reached around pH 5. A decrease in pH to 5.8 was also reported by Arhoun et al. [20] in the anaerobic digestion of strawberry residues. These authors also described that at low pH, the methane concentration also decreased and, finally, the biological activity ceased, as observed in the last stage of their study. Figure 2 shows how the alkalinity decreased when the pH dropped and increased again when the sodium bicarbonate buffer was added to the reactors. The decreasing trend varied from 3000 mg of CaCO_3_/L to 500 mg of CaCO_3_/L in the different stages. As was demonstrated throughout the experiment, buffer must be added for this type of substrate to avoid reactor acidification, given its acidic character. The values of the alkalinity in the reactors were below 3000 throughout the experiment, while it is recommended to achieve values of around 5000 to ensure the adequate stability of the process, as reported in other studies [22].

Figure 2 also shows the CODs and total VFA variation throughout the digestion time for the different OLRs assayed. The CODs and VFAs are important parameters in the anaerobic digestion processes for indicating the performance of the hydrolysis/acidogenesis stages of these processes [23]. In stage 1, i.e., 0–56 days and OLR = 1 g of VS/(L·d), the CODs and total VFAs increased to values of 4000 mg of O_2_/L. This high increase in CODs and total VFAs caused a decrease in pH and alkalinity, and thus, in turn, methane production was unstable in Stage 1 with OLR = 1 g of VS/(L·d). Therefore, it was decided to decrease the organic loading rate to a value of 0.5 g of VS/(L·d). In Stage 2, i.e., 57–107 days and OLR = 0.5 g of VS/(L·d), CODs and total VFAs showed more stable values, reaching mean values of around 2372 ± 165 mg of O_2_/L and 2139 ± 255 mg of O_2_/L, respectively. In Stage 3, i.e., 108–121 days, the OLR was again increased to 1 g of VS/(L·d) since in Stage 2, the reactor was stabilized, resulting in CODs and total VFA mean values of around 2793 ± 194 mg of O_2_/L and 2418 ± 219 mg of O_2_/L, respectively, very similar to those reached in Stage 2. As can be seen in Figure 2, the pH and alkalinity decreased again, and the reactors did not support an OLR = 1 g of VS/(L·d), so it was decided not to wait for three times the HRT with this OLR and to quickly change to a lower load before the reactors destabilized. In Stage 4, i.e., 122–284 days and OLR = 0.75 g of VS/(L·d), the CODs and total VFAs presented mean values of around 3741 ± 1504 mg of O_2_/L and 3131 ± 893 mg of O_2_/L, respectively. In Stage 4, it was decided to maintain the mentioned OLR for a longer time to assess the process stability. In this case, it was observed that it was necessary to provide a buffer for keeping the stability. Finally, in Stage 5, i.e., 285–310 days, the OLR was again increased to 1 g of VS/(L·d) to finish the study. It was observed that at this load, the reactors were not stable; the CODs and total VFAs presented mean values of around 12,108 ± 1412 mg of O_2_/L and 5200 ± 8 mg of O_2_/L, respectively. The increases in COD and VFAs were produced by the overloading and saturation of the reactors when exceeding the OLR limit. A similar trend was observed when the OLR increased from 1 to 2 g of VS/(L·d) in the semi-continuous anaerobic digestion of olive mill solid waste [22]. Table 3 also shows that in all the stages, more than 80% of the total solids corresponded to VS, except in the last stage, in which this value was only 60% of the VS.

**Figure 3:**
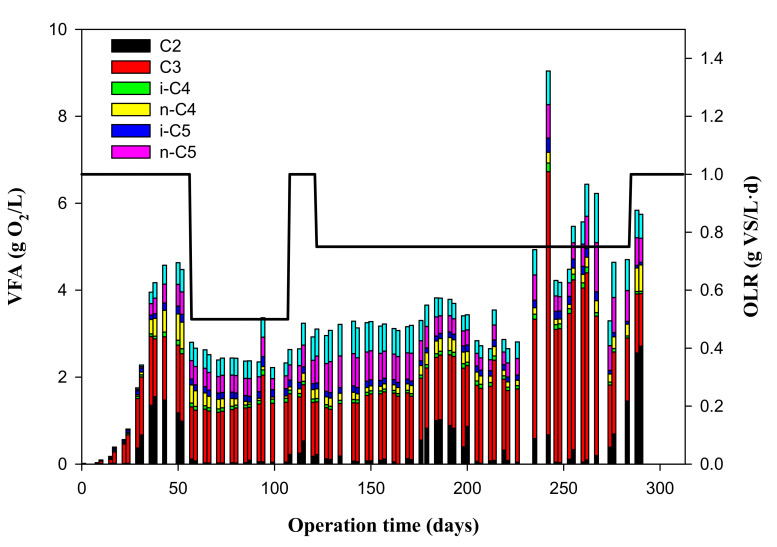
Variation of the VFA values, with their standard deviations, under the different organic loading rates (OLRs, line bold) during the experimental time.

The individual VFAs are shown in Figure 3. These were determined two times per week and are expressed in mg of O_2_/L. In Stage 1, i.e., 0–56 days and OLR = 1 g of VS/(L·d), C2 and C3 increased to values up to 1500 mg of O_2_/L, n-C4 and n-C5 increased to values up to 600 mg of O_2_/L, and i-C4 and i-C5 were also augmented to values up to 150 mg of O_2_/L. In Stage 2, i.e., 57–107 days and OLR = 0.5 g of VS/(L·d), individual VFAs can also be observed, although the process was much more stable, showing a mean C3 value of 1298 ± 211 mg of O_2_/L and C2, n-C4, i-C4, n-C5, and i-C5 concentrations below 500 mg of O_2_/L. In Stage 3, i.e., 108–121 days, it was decided again to raise the OLR to 1 g of VS/(L·d) since in Stage 2, the reactor was stabilized. The individual VFAs in Stage 3 presented a mean C3 value of 1306 ± 65 mg of O_2_/L, and a mean n-C5 value of 444 ± 90 mg of O_2_/L, with C2, n-C4, i-C4, and i-C5 below 500 mg of O_2_/L. In Stage 4, i.e., 122–284 days and OLR = 0.75 g of VS/(L·d), the individual VFAs presented a mean C3 value of 2189 ± 1551 mg of O_2_/L, and mean n-C5 value of 760 ± 1243 mg of O_2_/L, with C2, n-C4, i-C4, and i-C5 below 500 mg of O_2_/L.

### 3.3. Variation of the Concentration of Phenolic Compounds with Organic Loading Rate throughout the Experimental Time

**Figure 4:**
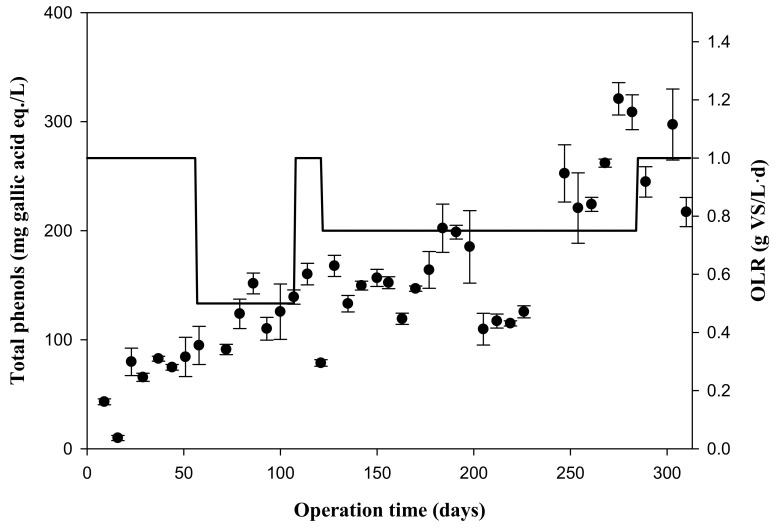
Variation of the total phenol concentrations, with their standard deviations, with the organic loading rate (OLR, bold line) throughout the experimental time.

Phenolic compounds were determined once per week and are expressed in mg of gallic acid equivalents/L (Figure 4). The concentration of phenolic compounds increased in Stage 1 and at the beginning of Stage 2 until reaching an average value of 119 ± 22 mg of gallic acid equivalents/L. As shown in Figure 4, the phenolic compounds remained approximately constant until the end of Stage 4, where they began to increase again due to the instability of the reactors. The highest concentration of phenolic compounds was reached in Stage 5, with 271 ± 37 mg of gallic acid equivalents/L, when the reactors are unstable due to the excess of added organic load (Figure 4). Phenolic compounds at a certain concentration can inhibit the activity of anaerobic microorganisms, affecting the microbial growth [24,25]. However, the phenolic concentrations found in these assays were always lower than those considered inhibitory for anaerobic digestion processes. The increase in phenols was similar to that found by Serrano et al. [21], who reported that exploiting the high solubilization capacity of phenols in an acidogenic fermentation at pH 4 is a promising method for the recovery of phenols from strawberry waste [21].

### 3.4. Economic Assessment

The minimum sales price of the phenolic compound extract was calculated for zero net profit, being EUR 0.610/g of gallic acid equivalents for economic viability at an OLR of 0.5 g of VS/(L·d). In a recent study by Trujillo-Reyes et al. [11], in which two strawberry extrudates from two different campaigns were hydrothermally treated at 150 °C for 60 min, similar results were obtained, i.e., EUR 0.556 and 1.23/g of gallic acid equivalents. However, the steam consumed in the steam-explosion treatment of SE, 6.71 kg of steam/kg of SE, was much higher than the values reported in the study by Trujillo-Reyes et al. [11], i.e., 1.93 and 0.44 kg of steam/kg of SE. Moreover, the extracted phenolic compounds in both studies were also similar, 0.90 g of gallic acid equivalents/kg of extrudate from steam-exploded SE in the present work versus 0.88 g of gallic acid equivalents/kg of extrudate in Strawberry Extrudate 1 in the study by Trujillo-Reyes et al. [11]. According to these data, the high steam consumption was not compensated for by the low methane production yield at an OLR of 0.5 g of VS/(L·d); however, the high recovery and a low sales price for the phenolic compounds made the economic assessment positive.

## 4. Conclusions

The semi-continuous anaerobic digestion of de-phenolised strawberry extrudate previously treated thorough steam explosion was stable at an OLR of 0.5 g of VS/(L·d), with a specific methane production rate of 135 ± 11 mL of CH_4_/(g of VS d). For OLRs higher than 0.5 g of VS/(L·d), the increase in soluble organic matter caused an overloading of the reactors. This overloading entailed an increase in the concentration of propionic acid to 1306 mg/L. Regardless of the OLR, the concentration of phenolic compounds was always lower than the inhibition limits. Economic analysis showed that the combination of the steam explosion, recovery of phenols, and anaerobic digestion with a low OLR, i.e., 0.5 g of VS/(L·d), under stable conditions, generated a low sales price for the phenol extract, i.e., EUR 0.610/g of gallic acid equivalents, making for a positive economic assessment. Further research about the role of the individual phenolic compounds in SE during the anaerobic digestion would be interesting for determining their inhibitory potential.

## Figures and Tables

**Table 1:** Physicochemical characterization of the strawberry extrudate (SE) and of different phases resulting from hydrothermal treatment and extraction of phenolic compounds.

	SE	First Steam-Explosion Treatment			Second Steam-Explosion Treatment		
	SE	SP	LP	DLP	SP	LP	DLP
**pH**	3.7 ± 0.1	4.5 ± 0.1	4.0 ± 0.1	4.1 ± 0.1	3.9 ± 0.1	3.9 ± 0.1	3.9 ± 0.1
**TS (SE)**	142 ±1	97 ± 2	48 ± 1	40 ± 1	92 ± 2	45 ± 1	26 ± 1
**VS (g/kg of SE)**	136 ± 1	93 ± 2	48 ± 1	40 ± 1	88 ± 2	41 ± 1	24 ± 1
**COD_t_ (g of O_2_/kg of SE)**	200 ± 6	132 ± 2	61 ± 2	51 ± 2	171 ± 6	55 ± 6	34 ± 3
**COD_s_ (g of O_2_/kg of SE)**	47 ± 1	9 ± 1	64 ± 2	54 ± 2	2 ± 1	51 ± 2	39 ± 1
**Total phenols** **(g of gallic acid eq./kg of SE)**	1.4 ± 0.1	0.7 ± 0.1	1.3 ± 0.1	0.4 ± 0.1	4.4 ± 0.1	0.5 ± 0.1	0.1 ± 0.1
**Total sugars (g of glucose eq./kg of SE)**	2.1 ± 0.1	0.5 ± 0.1	24.9 ± 0.1	21.8 ± 0.1	6.8 ± 0.1	25.6 ± 0.1	18.9 ± 0.1

SE, strawberry extrudate; SP, solid phase; LP, liquid phase; DLP, de-phenolized liquid phase; TS, total solids; VS, volatile total solids; COD_t_, total chemical oxygen demand; COD_s_, soluble chemical oxygen demand.

**Table 2:** Experimental design.

Experimental Stage	Duration	Fed Substrate	OLR (g of VS/(L·d))
Bio-stimulation	16 days	SS	1
Adaptation	4 days	SS:SE 75:25, in VS	1
	13 days	SS:SE 50:50, in VS	1
	12 days	SE 100, in VS	1
Stage 1	56 days (0–56)	SP+DLP	1
Stage 2	50 days (57–107)	SP+DLP	0.5
Stage 3	13 days (108–121)	SP+DLP	1
Stage 4	62 days (122–284)	SP+DLP	0.75
Stage 5	26 days (285–312)	SP+DLP	1

SS, Synthetic solution with glucose (50 g/L) and sodium acetate (25.2 g/L).

**Table 3:** pH, alkalinity, TS, VS, CODs, total volatile fatty acids (VFAs), total phenols, methane production rates, and biodegradability under the different organic loading rates (OLRs).

	Stage 1	Stage 2	Stage 3	Stage 4	Stage 5
OLR (g of VS/(L·d)	1	0.5	1	0.75	1
Days	0–56	57–107	108–121	122–284	285–310
pH	6.3 ± 0.6	6.1 ± 0.3	6.2 ± 0.3	6.0 ± 0.7	6.9 ± 0.5
Alkalinity (mg of CaCO_3_/L)	1500 ± 660	915 ± 177	1172 ± 281	1717 ± 237	8032 ± 333
TS (mg of TS/L)	9588 ± 2840	10,842 ± 866	12,746 ± 1318	14,990 ± 4963	26,163 ± 2636
VS (mg of VS/L)	7658 ± 2673	9412 ± 950	11,200 ± 1541	12,848 ± 3454	15,581 ± 324
CODs (mg of O_2_/L)	1799 ± 1394	2372 ± 165	2793 ± 194	3741 ± 1504	12,108 ± 1412
Total VFA (mg of O_2_/L)	1838 ± 1752	2139 ± 255	2418 ± 219	3131 ± 893	5200 ± 8
Total phenols (mg of gallic a. eq./L)	63 ± 27	119 ± 22	119 ± 58	182 ± 63	271 ± 37
Methane production (mL of CH_4_/g of VS d)	124 ± 46	135 ± 11	130 ± 10	129 ± 45	64 ± 43
Biodegradability of CH_4_ (%)	10 ± 3	9 ± 2	16 ± 1	12 ± 4	7 ± 2
Biodegradability of VS (%)	70 ± 4	53 ± 1	51 ± 1	60 ± 10	48 ± 9
